# Application of in-line imaging technology in preparation of Ziegler–Natta catalysts for propylene polymerization

**DOI:** 10.1039/d6ra02038k

**Published:** 2026-04-14

**Authors:** Sihan Li, Zhan Shi, Lian Yan, Wei Cen, Jiahui Shao, Meiyan Fu

**Affiliations:** a Division of Polypropylene Research, SINOPEC (Beijing) Research Institute of Chemical Industry Co., Ltd. No. 14, N. 3^rd^ Ring Road East Beijing 100013 China lisih.bjhy@sinopec.com fumy.bjhy@sinopec.com

## Abstract

Morphological control is essential in achieving high performance in polypropylene production, which can be attributed to the morphology replication effect of MgCl_2_-supported Ziegler–Natta catalysts (ZNCs). Although well-established models, such as the “sea urchin” crystallization theory developed by Chang *et al.*, have successfully elucidated the internal crystallographic orientation, the mechanism of morphological formation of the hierarchical secondary particles still remains unclear. In our study, we use *in situ* imaging technology to observe the formation of ZNC particles in real-time in a synthesis environment. The following thermally driven crystallization mechanism was revealed in the experiment: liquid–liquid phase separation, initial nucleation and aggregation, crystals growth and solidification. From the SEM analysis, the morphology of the particle is unique botryoidal, or grape-like, and consists of 5 to 30 tightly fused primary units (3–8 µm) that assemble into secondary particles (15–25 µm). The control experiment shows that the invasive *in situ* probe neither interferes with the formation of the catalyst nor affects the types and distribution of the active sites, which enables us to observe the actual formation process of the catalyst particles. Finally, the propylene polymerization experiment produced polymer particles that perfectly replicated the botryoidal structure of the catalysts, achieving a particle size of 3 mm. The activity and stereoregularity control capabilities of the prepared catalysts are comparable to the commercial catalysts.

## Abstract 2

Monitoring the dynamic growth of Ziegler–Natta catalyst particles through in-line imaging technology.

## Graphical abstract

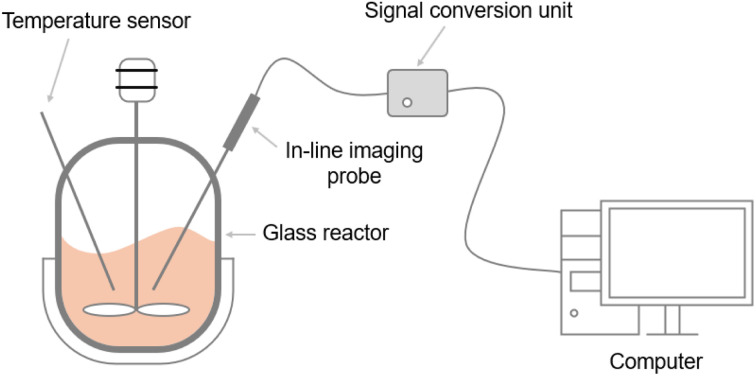

## 1. Introduction

Polyethylene (PE) and polypropylene (PP) represent the two most predominant commercial polyolefins. In 2024, the share percentage for the production of PE was around 26%, whereas for PP it was around about 20%.^1^ Within this sector, over 95% of industrial isotactic polypropylene is manufactured using heterogeneous Ziegler–Natta catalysts (ZNCs). The widespread use of ZNCs can be mainly attributed to their ability to facilitate the continuous production process, better operability, and precise control of the morphology of the final polymer products.^2,4^ Considering the fact that the morphology of the final polymer products is mainly dictated by the physical properties of the catalyst, understanding the evolution of catalyst structure is of paramount importance.^3^

Due to the morphology replication effect,^5,6^ the physical properties of the ZNCs play a crucial role in controlling the morphology of the formed polymer. In this regard, the achievement of precise control over the morphology of the catalyst particles is a key goal in both scientific research and industrial applications. Three main methodologies, namely ball-milling (physical method), recrystallization,^7^ and chemical precipitation, are commonly used for the synthesis of MgCl_2_-supported ZNCs. The mechanism of the crystallization of the ZNCs has been extensively investigated over the past decades. One notable contribution was offered by Chang *et al.*,^8^ who proposed the “sea urchin” model of crystallization, which was used to describe the radial growth and internal orientation of MgCl_2_ crystallites. The model shows that the supporting particles are hierarchical agglomerates of multiple primary particles that are densely packed with elongated MgCl_2_ crystal rods. Within the crystal rods, the Cl–Mg–Cl crystal layers are oriented primarily perpendicular to the longitudinal axis; consequently, the *c*-axis of the MgCl_2_ crystal lattice is parallel to the rod length. This particular structural arrangement is of paramount importance, as it maximizes the exposure of the lateral surfaces, where the active catalytic centres will generate following the treatment with TiCl_4_. Taniike and co-workers^9^ systematically investigated the compositional and structural evolution of ZNCs during their synthesis from a Mg(OEt)_2_ precursor, utilizing a combination of X-ray total scattering and IR spectroscopy. By analyzing five intermediate samples extracted from different synthesis stages, they demonstrated that the contact between the Mg(OEt)_2_ and TiCl_4_ triggers an instantaneous conversion of the Mg–O bonds to Mg–Cl bonds. This process yields initial MgCl_2_ seeds characterized as Cl–Mg–Cl monolayers with lateral dimension below 2 nm. At this stage, a significant amount of Ti remains physiosorbed as 4-fold-coordinated TiCl_*x*_(OEt)_4−*x*_ species. Subsequent thermal treatment facilitated the transformation of these physiosorbed species into chemisorbed 6-fold-coordinated TiCl_*x*_(OEt)_4−*x*_. Crucially, the introduction of an internal donor promotes a substantial reconstruction and growth of the MgCl_2_ seed, exposing more catalytically active lateral surfaces and reaching a crystallite size comparable to the final catalyst. However, although these studies provide profound insights into micro-crystallinity, they still leave a gap regarding the mechanistic origin and structural evolution of the particle morphology at the macroscopic level.

Due to the high sensitivity of ZNCs to air and moisture, as well as the corrosive environment of excess TiCl_4_, it has been difficult to utilize many conventional *in situ* characterization techniques, such as static/dynamic light scattering, optical microscopy and X-ray scattering for real-time process monitoring. Patience *et al.* developed one of the earliest image analysis-based process monitoring and optical imaging systems.^10^ In recent years, the image recording and processing tools have been developed and are now widely used in the crystallization process of the pharmaceutical and food industries.^11,12^ These techniques are suitable for monitoring crystal shape and have now become a powerful tool in process analytical technology (PAT).^13–18^ Building upon these advancements, the present work extends this methodology to the more challenging environment of ZNCs synthesis.

In the current work, the formation and dynamic growth of ZNCs particles were monitored by using *in situ* imaging technology during the synthesis process. Based on the real-time observation and multi-scale characterization, including X-ray diffraction (XRD), scanning electron microscopy (SEM) and N_2_ adsorption–desorption isotherms, a general mechanism of the formation of ZNCs is proposed. Moreover, the propylene polymerization test verifies the relationship between the morphology of the catalyst and its replication behaviour.

## 2. Materials and methods

### 2.1 Materials

All the materials and catalysts used in this work were manipulated in glovebox or in an inert atmosphere of dry N_2_. MgCl_2_ provided from Sinopec Catalyst Co. Ltd (Beijing, China) and used as received. TiCl_4_, toluene, and *n*-hexane were supplied by Aladdin Scientific Co., Ltd (Shanghai, China), triethylaluminium (TEA) 1.0 M solution in *n*-hexane, tributyl phosphate, epichlorohydrin, phthalic anhydride and cyclohexylmethyldimethoxysilane (CMDMS) were purchased from J&K Scientific Co., Ltd (Beijing, China). All chemicals were used as received, unless mentioned otherwise. Toluene, *n*-hexane, tributyl phosphate and epichlorohydrin were dried and stored over molecular sieve 4 Å.

### 2.2 Preparation of the catalyst

The catalysts were synthesized as described previously.^19^ The pre-catalysts are prepared by dissolving a mixture of complexes of anhydrous MgCl_2_ with epichlorohydrin and tributyl phosphate in toluene at 50 °C, cooling the solution to −20 °C, and removing the organic components from the MgCl_2_ complexes with excess of TiCl_4_. Heating the solution slowly to 80 °C results in precipitation of solid support particles. The support is contacted with internal donor at certain temperature, activated with TiCl_4_ in toluene solution at certain temperature and finally washed thoroughly with toluene and hexane. The procedures for catalyst preparation and sampling are shown in Scheme S1.

### 2.3 Propylene polymerization reaction

Polymerization tests were performed with the produced catalyst particles to evaluate their activity and the morphology of the corresponding polymer powder. To this purpose, bulk polymerization of propylene was carried out. Further operating conditions are summarized in Table 1.

**Table 1:** Summary of polymerization conditions used in the catalytic tests

Propylene volume (L)	2.2
Catalyst (mg)	10.0
Co-catalyst TEAl (mmol)	5.0
External donor (mmol)	0.1
Propylene/H_2_ (mol mol^−1^)	146
*T* (°C)	70.0
*t* (min)	60
Stirring speed (rpm)	150

Propylene bulk polymerizations were carried out in a stirred 5 L tank reactor. TEAl was used as co-catalyst and scavenger, CMDMS was used as the external donor. Designated amounts of TEAl, external donor, catalyst powder and H_2_ were successively introduced into the reactor. Liquid propylene was added to start the reaction. After the reaction, discharge residual propylene from the reactor vessel and purge the reactor three times with N_2_, the resulting polypropylene was obtained without further purification.

### 2.4 Characterization

#### SEM micrographs

The micrographs were collected on a SU8600 (HITACHI). In order to avoid contact with ambient air, the samples were plated on a sample holder and quickly moved into the Au sputtering equipment. The micrographs were acquired with an acceleration voltage of 5 kV. Before the SEM measurements, the sample was subjected to Au sputtering for 3 min.

#### Powder X-ray diffraction

XRD was performed on a Bruker D8 Advance diffractometer (Germany) with Cu-Kα radiation. The diffraction patterns were recorded with a 0.02 scanning step at an angle range of 2° from 5 to 90° and accumulation period of 5 s at every point.

#### Particle size measurements

The particle size measurement after the catalyst preparation was performed by a laser diffraction instrument, Mastersizer 3000 (Malvern), inside the drybox. 10 mg catalyst samples were dispersed in 250 mL pre-dried and degassed hexane in hydro dispersion unit. The particle size is expressed with *D*_10_, *D*_50_ and *D*_90_, which correspond to the particle size at 10%, 50% and 90% of the cumulative volume-base distribution. A relative span factor (RSF) was calculated based on eqn (1):1
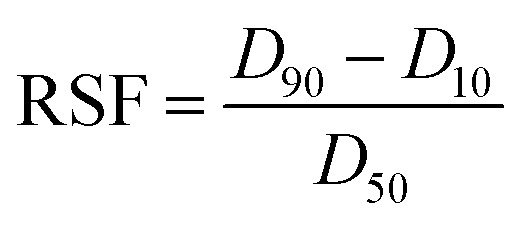


#### Isotacticity index (I.I.)

I.I. is measured following the international standards ISO 9113:2019.

#### Melt flow rate (MFR)

The MFR of polypropylene was tested on Göttfert mi40 (Germany) at 230 °C, under the pressure of 2.16 kg, following the international standards ISO 1133-1:2022.

#### Chemical composition

Ti was measured by spectrophotometry; Mg was measured by EDTA titration at a pH of 10 against Eriochrome Black T. (for details see SI).

#### Differential scanning calorimetry (DSC)

DSC was performed using a TA DSC Q2000 differential scanning calorimeter that was calibrated using high purity indium at a heating rate of 10 °C min^−1^. Melting points were determined from the second scan at a heating rate of 10 °C min^−1^, following a slow cooling rate of 10 °C min^−1^ to remove the influence of thermal history.

#### N_2_-adsorption–desorption isotherm

The pore structures of the samples were measured by Micrometrics ASAP 2460 Surface Area and Porosity Analyzer at 77 K. The BET surface area (*S*_BET_) was analyzed by Brunauer–Emmett–Teller theory. Pore size distributions were estimated with the Barrett–Joyner–Halenda (BJH) method^20^ using the Harkins and Jura standard isotherm. The total pore volume was determined according to the amount adsorbed at a relative pressure *P*/*P*_0_ of 0.95.

## 3. Results and discussion

### 3.1 Catalyst morphology

**Fig. 1:**
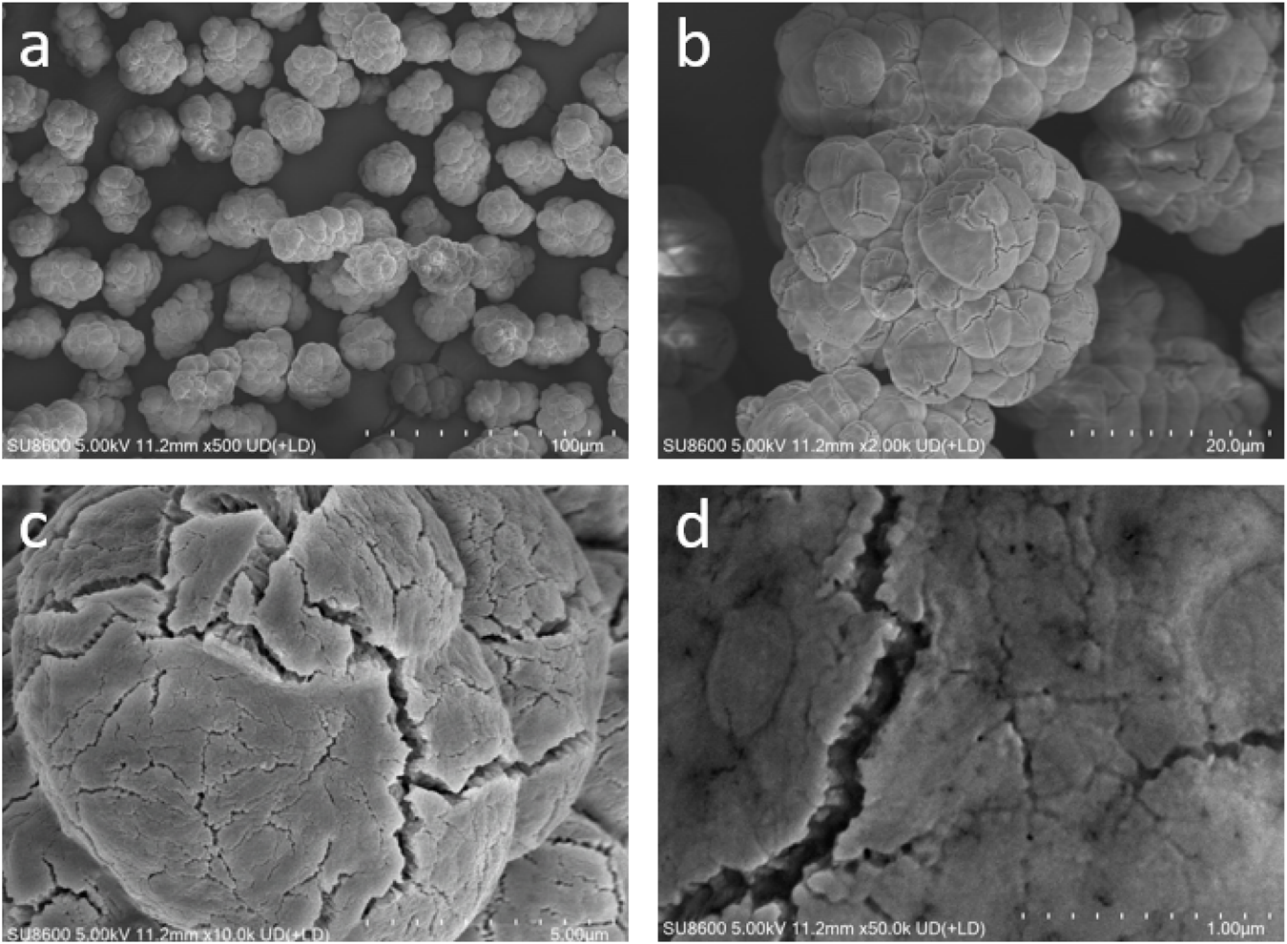
SEM micrographs of Cat1 particles collected at magnification 500× (a), 2000× (b), 10 000× (c) and 50 000× (d).

The morphology of the synthesized catalysts was characterized using scanning electron microscopy (SEM), as shown in Fig. 1. At a low magnification of 500×, the catalyst particles exhibited a distinct botryoidal morphology with smooth contours and the absence of sharp edges. The particle size distribution is relatively narrow, with the majority of secondary particles having sizes in the range of 20–30 µm (Fig. 1a). It is clear at medium magnification (2000×) that these secondary particles are made of hierarchical structures composed of 5–30 closely merged primary spherical particles of different sizes, with the diameter of primary particles ranging from 3–8 µm (Fig. 1b). Notably, due to this extensive inter-particle fusion, a large part of surface area of primary particles is shielded. This structural feature that likely governs the subsequent polymerization kinetics, such as limited active sites exposure^21,22^ or monomer diffusion.^23,24^

Observations using high-magnification SEM at a magnification ranging from 10 000–50 000× are essential for providing insights into the microstructure of primary particles. The primary particles have a distinct grainy texture with many micro-cracks (Fig. 1c). The grains on surface are terminations of rod-like crystalline structures that develop from inside and grow towards the periphery (Fig. 1d).^7^ It should be noted that this hierarchy morphology is quite different from that which is normally seen in anhydrous MgCl_2_, which tends to be overlapping platelets. The formation of the hierarchical catalyst morphology can be explained by a process of synchronized radial crystallization.^25,26^ Subsequently, fibrous MgCl_2_ grows out from these nucleation centers with a constant radial velocity. This result in the formation of primary units that have spherical shape. As these growing units continue to increase in size in confined medium of MgCl_2_ solution phase, they eventually make contact with neighbouring units and finally merge with them.

This type of overlap also enables the process of solid-state fusion, where the radial rods of adjacent particles lock into place, effectively fusing the individual primary particles into the observed botryoidal aggregates. This process is unlike the more traditional ZNCs, prepared *via* alternative crystallization routes, which often manifest as loose aggregates sub-micron MgCl_2_ particles.^27,28^

This growth pattern ensures that the secondary particles have high structural integrity, as indicated by the “fusion zones” acting as mechanical bridges for the primary spherical particles. This arrangement enhances the mechanical robustness of the particles and reduces premature fragmentation during subsequent catalyst processing steps and polymerization.^29–32^

### 3.2 *In situ* imaging monitor catalyst formation

*In situ* imaging techniques were utilized to monitor the morphological evolution of the catalyst particles during the synthesis process. The experimental setup consisted of a reaction system and an imaging system. The synthesis was carried out in a 1 L jacketed glass reactor from Büchi (Germany), which is equipped with a precise thermal control system with a temperature range from −30 to 150 °C. The imaging assembly consisted of an in-line imaging probe (Pharmavision Nanosonic Technology Ltd), a signal conversion device and a computer. In order to withstand the extremely corrosive environment caused by the use of TiCl_4_, the terminal probe with the special slot for sensing was made of Hastelloy. The captured image has a resolution of 2448 × 2048 pixels, the size of the monitoring area is 500 × 500 µm. Details of the imaging probe are shown in Fig. S1. After all the TiCl_4_ has been added to the solution, the imaging probe starts to record the picture every 15 seconds until the temperature reach to aging temperature (Scheme S1a and b).

**Fig. 2:**
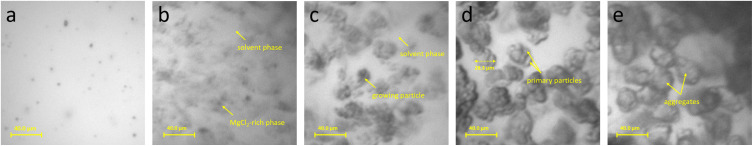
In-line imaging observations results during Cat1 synthesis process between stage (a) to (b) (Scheme S1) from different temperature, (a) (before adding TiCl_4_, −20 °C), (b) (after adding TiCl_4_, −20 °C), (c) (10 °C), (d) (30 °C), (e) (80 °C).

Fig. 2a depicts the initial state of the precursor MgCl_2_ solution, the black spots were confirmed to be the partially undissolved MgCl_2_ (around 2% of the initial amount of MgCl_2_ added, Fig. S2). The slow addition of TiCl_4_ triggers liquid–liquid phase separation (Fig. 2b). In this stage, the MgCl_2_-rich phase is dispersed in the continuous TiCl_4_ phase; however, the boundaries are not clearly defined due to the continued phase separation and the lack of an emulsifier. The state of the solution remained virtually unchanged compared to that observed before the addition of TiCl_4_, no precipitation of particles was observed, and the solution had a slight turbidity (Fig. S3).

**Scheme 1:**
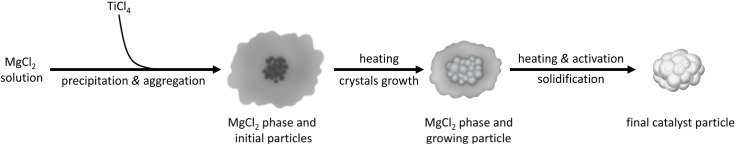
Scheme showing the catalyst particle formation process, which involves the initial nucleation and aggregation, the crystals growth and solidification.

As the temperature rises, *in situ* crystallization begins within the MgCl_2_-rich phase (Fig. 2c), which is characterized by the significant opacification (darkening) of the dispersed phase. Subsequently, these initial crystallites go through an agglomeration process, with increasing temperature, the existing aggregates act as nuclei to initiate a growth stage within the MgCl_2_ phase. Upon attaining 30 °C, the boundaries of the interface are well defined, and the formation of particles with hierarchical structural features can be observed (Fig. 2d). It is worth noting that at this point, the particle sizes no longer show any significant change. As the temperature rises, the particles undergo further solidification, yielding well-defined botryoidal particles (Fig. 2e) that corroborate the morphological observations obtained *via* SEM. Through the direct observation of the transition from liquid–liquid phase separation to the formation of solid particles, we suggest that the temperature exerts a significant influence on the entire reaction process (Scheme 1).

**Fig. 3:**
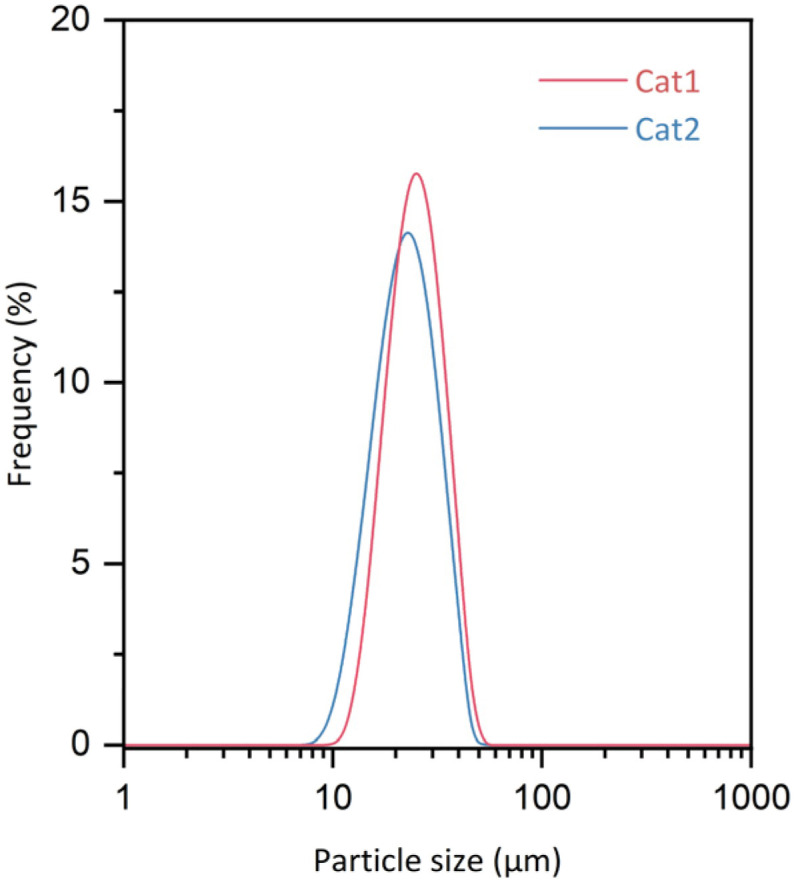
Particle size distribution of Cat1 and Cat2.

The morphology of the catalyst, as it forms in the synthesis of ZNCs, is affected not just by the stoichiometry of the reactants, but also by the reaction conditions used. For example, Ko and co-workers have shown that the configuration of the baffles used in the reactor has a considerable influence on the catalyst's structure and morphology.^33^ As the invasive in-line imaging probe may function as a baffle, potentially altering the fluid dynamics and experimental outcomes, a control experiment was conducted. A control catalyst (Cat2) was synthesized under identical conditions but in the absence of the in-line probe. SEM analysis has also confirmed that Cat2 has the same botryoidal morphology and primary spherical particles as the probe-monitoring sample (Fig. S4). Moreover, both catalysts exhibited a similar particle size distribution, which is narrow (Fig. 3), with a median diameter of approx. 25 µm (*D*_50_). These results suggest that the in-line probe does not affect the mechanism of particle growth or the overall structure.

### 3.3 Crystalline structure

**Fig. 4:**
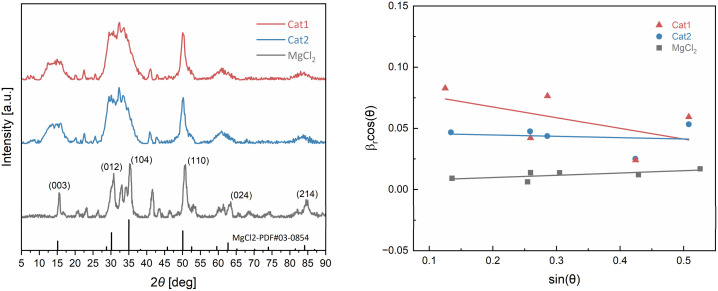
Power X-ray diffraction patterns (left) and Williamson–Hall plot (right) of mechanically activated MgCl_2_, Cat1 and Cat2.

The powder X-ray diffraction (PXRD) patterns of the raw MgCl_2_ and catalyst samples are illustrated in Fig. 4. Mechanically activated MgCl_2_ (prepared *via* 30 hours of ball-milling) exhibits a mixture of cubic and hexagonal packing of the Cl–Mg–Cl layers, as identified by the characteristic peaks of the α-MgCl_2_ in the 2*θ* range of 30–35°. The (012) and (104) diffraction peaks, which correspond to the body-diagonal direction, show significant merging and broadening along with a small shift. Such behaviour suggests the presence of the stacking disorder along the *c*-axis and the reduced crystallite size for the MgCl_2_. The reduced crystallite size and stacking fault density benefit the dissolution of MgCl_2_ in toluene with the presence of phosphates.^34^

In addition to broadening induced by grain size, the broadening is also significantly caused by crystal lattice distortion (micro-strain).^35^ Here, we employed the Williamson–Hall eqn (2)^36^ to investigate micro-strain within the samples:2
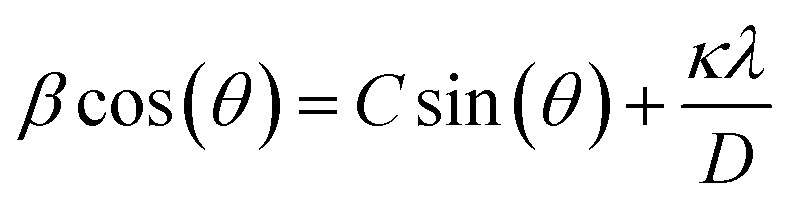
Here, *β* is the full width at half maximum (FWHM), and *D* is the crystalline size. *λ* is used to represent the wavelength of the CuKα radiation and *κ* is the shape factor (0.9). According to the Williamson–Hall equation, the total broadening of the XRD peaks is due to the combined effects of the crystallite size and lattice strain (including stacking disorder, represented by *C* in the first part of the equation). Consequently, the lattice strain within the crystallites can be quantitatively determined by constructing a plot of *β* cos(*θ*) against sin(*θ*) and using the slope of the resulting linear regression fit. As shown in Fig. 4, the synthesized catalysts have negative slopes (Cat1 = −0.088, Cat2 = −0.011), which is in contrast to the positive slope of the pristine MgCl_2_ (0.018). This shift in the gradient from positive to negative suggests a pronounced structural disorder, and it is not the micro-strains that cause the broadening.^37^

Cat1 and Cat2 align with those of mildly ground MgCl_2_, their FWHM differs substantially, indicating distinct crystallite morphologies. The XRD pattern of both catalysts exhibits distinct δ-MgCl_2_,^38,39^ the broadening and merging in the 2*θ* range of 30–35°, indicating that the resulting catalyst exhibits smaller grain sizes and more stacking disorder. Moreover, the broadening and remaining intensity of the peaks at 2*θ* = 15° of the (003) planes, suggests more than 10 Cl–Mg–Cl layers exist.^40,41^

The diffraction peak positions of a key reflection are the (104) plane, which is oriented at an oblique angle relative to the Cl–Mg–Cl layers, thus the crystallite sizes for both catalysts were estimated from the (104) peak to be in the range of 20–25 Å using the Scherrer equation,^42^ which is significantly smaller than the average diameter of the MgCl_2_ rods (100–200 Å). This difference suggests that the macroscopic rod-like structure is not a single crystal but rather composed of highly disordered nanocrystalline domains. The significant peak broadening, as well as the small, estimated crystallite size, serve as compelling evidence of the presence of considerable stacking disorder in the MgCl_2_ framework. These disorders play an essential role in creation of suitable binding sites for strong TiCl_4_ adsorption.^43^

This elevated state of disorder is thought to create an abundance of coordinatively unsaturated surface environments, which facilitate the efficient incorporation of Ti active centres and the coordination of internal electron donors. Consequently, the modified MgCl_2_ matrix is an excellent foundation for the formation of highly active catalytic complexes.

### 3.4 N_2_ adsorption–desorption isotherm

**Fig. 5:**
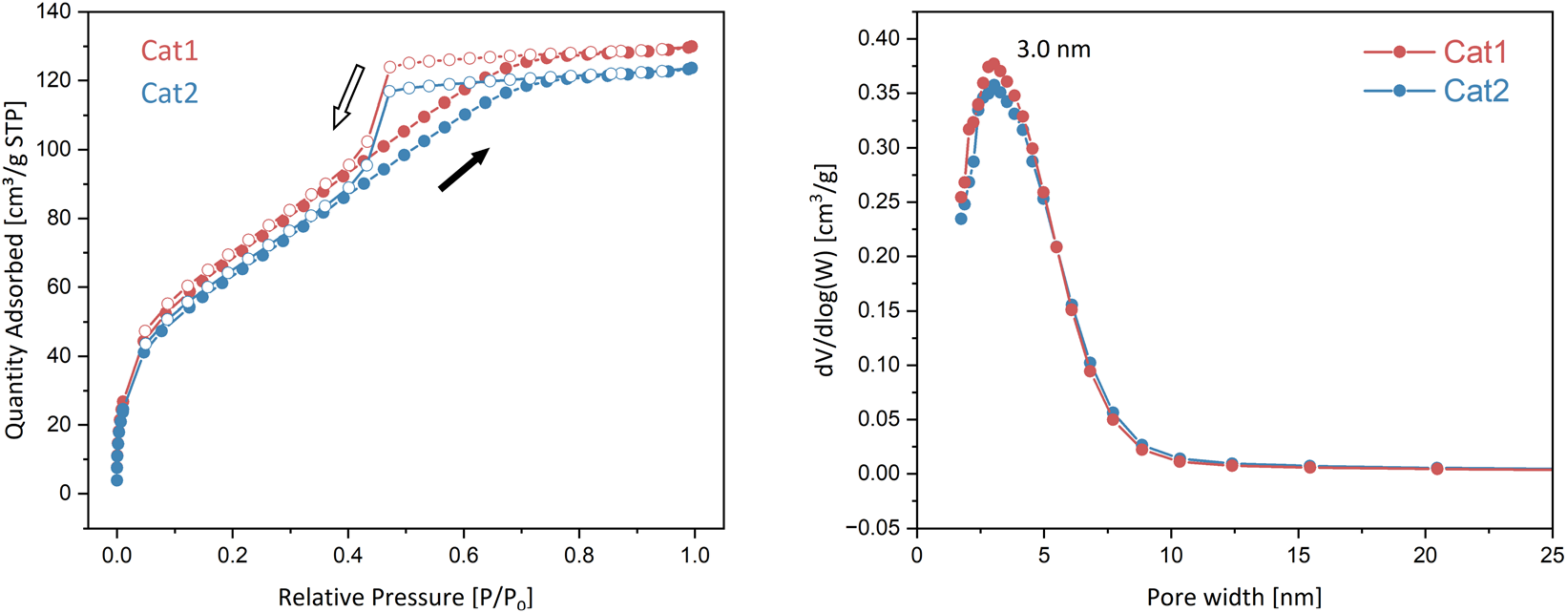
N_2_ adsorption–desorption isotherms and pore size distribution profile of Cat1 and Cat2 (for left figure, solid dots: adsorption; open dots: desorption).

The N_2_ adsorption–desorption isotherms were performed to characterize the porosity of the resulting catalysts. The BET surface area (*S*_BET_), external surface area (*S*_ext_) and total pore volume (*V*_total_) are summarized in Table S1. Both catalysts exhibit moderate *S*_BET_ values of 259.9 m^2^ g^−1^ (Cat1) and 239.9 m^2^ g^−1^ (Cat2), consistent results were obtained for *V*_total_, with values of 0.19 and 0.18 cm^2^ g^−1^ for Cat1 and Cat2, respectively. Fig. 5 shows that N_2_ adsorption–desorption isotherm of two catalysts were of semi-(IUPAC) type IV(a) with an inclined mound shape hysteresis classified as semi-IUPAC H2 according to the literatures,^44,45^ this confirms the predominantly mesoporous nature of the catalysts. By integrating the SEM observations and N_2_ adsorption–desorption characteristics with the crystallization model proposed by Chang *et al.*,^8^ it can be concluded that the catalysts possess a networked, slit-like geometry. This pore structure corresponds to the parallel arrangement of MgCl_2_ crystalline rods, where the interstitial areas form channels. Pore size distribution analysis further reveals that both catalysts have a major pore diameter close to 3 nm (Fig. 5). We suggest that the radial growth of rod-shaped MgCl_2_ crystallites is responsible for the complex mesoporous structure. This mesoporosity, narrow mesoporous distribution, combined with the networked architecture, provides an optimized environment for the diffusion of internal donors and the subsequent immobilization of Ti species, as well as a robust pathway for mass transfer of monomers during the early stages of polymerization.

### 3.5 Polymerization result

It has also been observed that the particles of the resulting polymer process an identifiable botryoidal structure, which is similar to the structure of the catalyst particles (Fig. S5), with an average particle size of about 3 mm. In addition to morphological consistency, the catalytic integrity was also evaluated to ensure that the presence of the in-line probe did not affect the formation of the active site. Bulk propylene polymerization tests were performed under the conditions summarized in Table 1, and both the probe-monitored catalyst (Cat1) and the control sample (Cat2) exhibited comparable catalytic activity of around 35 kg_PP_ g_cat_^−1^ and produced polymers with similar melt flow rates (Table 3). The resulting polypropylenes both exhibit similar isotacticity, indicating that the two catalysts have very similar types and quantities of active sites. These results, combined with the similar results from SEM and chemical composition (Table 2), clearly demonstrate that the chemical performance is unaffected by the in-line probe. Accordingly, it can be considered that the morphological evolution observed with the imaging probe is highly representative of the actual reaction environment and provides a reliable basis for further mechanistic studies.

**Table 2:** Summary of the particle size analysis, chemical composition and porosity results

Catalyst	Ti (wt%)	Mg (wt%)	*D*_10_ (µm)	*D*_50_ (µm)	*D*_90_ (µm)	RSF	BET surface area (g m^−2^)
Cat1	2.28	20.56	15.0	23.7	35.7	0.873	259.9
Cat2	2.17	19.96	17.8	26.4	38.5	0.784	239.9

**Table 3:** Summary of propylene polymerization results

Catalyst	Activity (kg_PP_ g_cat_^−1^)	I.I. (%)	*T*_m_ (°C)	MFR
Cat1	37.7	97.4	163.6	15.6
Cat2	35.1	97.7	161.0	18.1

## 4. Conclusions

In summary, this work provides a comprehensive understanding of the hierarchical morphogenesis of industrial Ziegler–Natta catalysts by bridging molecular-scale structural evolution with macroscopic particle formation. Firstly, we successfully captured the real-time dynamic evolution of catalyst particles under the synthesis conditions through the implementation of *in situ* imaging technology. Our findings suggest that the formation pathway of the catalyst follows the hierarchical assembly mechanism, the process begins with the phase separation, followed by isotropic crystal growth, driven by the synergistic effects of the TiCl_4_. Moreover, the systematic control experiments reveal that the invasive *in situ* probe has no noticeable effect on the precipitation/growth kinetics and the final chemical composition/morphology of the catalyst. This validation ensures that the real-time observations reported herein are highly representative of the authentic synthesis environment, providing a reliable foundation for catalysis optimizations. Furthermore, the microstructure of the catalyst particles was characterized by a combination of SEM, XRD and N_2_ adsorption–desorption isotherms. Through the use of *in situ* imaging for ZNCs synthesis, we have introduced a significant characterization approach that overcomes the limitations of static analysis and dynamic observation. This work provides valuable new insights into future studies on the formation mechanisms of catalysts and offers a powerful tool for the real-time observation and rational design of next-generation ZNCs.

## Author contributions

S. L.: conceptualisation, data curation, formal analysis, investigation, methodology, writing – original draft, writing – review & editing. Z. S., L. Y., W. C.: writing – review & editing. J. S.: data curation. M. F.: funding acquisition, supervision, writing – review & editing.

## Conflicts of interest

The authors declare that they have no conflicts of interests.

## Supplementary Material

RA-016-D6RA02038K-s001

## Data Availability

The authors declare that all data supporting the results reported in this study are available within the paper and the supplementary information (SI). Supplementary information is available. See DOI: https://doi.org/10.1039/d6ra02038k.
