# Supplementary material for: Application of in-line imaging technology in preparation of Ziegler–Natta catalysts for propylene polymerization
Source: RSC Adv. 2026 Apr 14;16(21):19449–56. doi: 10.1039/d6ra02038k (PMC13078406; doi:10.1039/d6ra02038k)
Supplement: RA-016-D6RA02038K-s001 [file RA-016-D6RA02038K-s001.pdf]

Supplementary Information (SI) for RSC Advances.
This journal is © The Royal Society of Chemistry 2026

Supplementary Information

for

Application of On-line Imaging Technology in Preparation of Ziegler-Natta Catalysts for Propylene Polymerization

Sihan Li,*[a] Zhan Shi,[a] Lian Yan,[a] Wei Cen,[a] Jiahui Shao,[a] Meiyan Fu*[a]

[a] Division of Polypropylene, SINOPEC (Beijing) Research Institute of Chemical Industry Co., Ltd., Beijing 100013, China

Chemical Composition Analysis

Mg content:

The Mg content was determined by complexometric titration with EDTA. To be specific, an accurately weighed catalyst sample (*m*, 0.05-0.06 g) is quantitatively transferred into a 50 mL volumetric flask, and the catalyst is dissolved in a solution of diluted sulfuric acid (1 mol/L). Next, 5.0 mL of the catalyst solution is transferred into a 250 mL Erlenmeyer flask, and to this solution, 10 mL of triethanolamine solution ($V_{triethanolamine}/V_{deionized\ water}$ = 1/2, which acts as a masking agent), 10 mL of an ammonia-ammonium chloride buffer (pH = 10), 6 drops of hydrogen peroxide (for eliminating interference from Ti species), and an appropriate amount of Eriochrome Black T indicator were added sequentially. The solution is titrated with a standard solution of EDTA (*c* = 0.02 mol/L) with an acid burette, and titration is continued until the solution changes color from wine-red (purple) to pure blue. The volume of EDTA solution used in titration (V_{EDTA}) is measured, and the content of Mg (ω) was calculated by the following equation:

$$\omega = \frac{V_{EDTA} \times c \times M_{Mg}}{m} \times 100\%$$

Ti content:

The Ti content was analyzed using UV-Vis spectrophotometry. To be specific, an accurately weighed catalyst sample (*m*, 0.02-0.05 g) is quantitatively transferred into a 50 mL volumetric flask, and the catalyst is dissolved in a solution of diluted sulfuric acid (1 mol/L). Using dilute sulfuric acid as the blank control, the absorbance reading of the blank solution containing dilute sulfuric acid was adjusted to 0. Subsequently, the initial absorbance of the catalyst sample reading was recorded as A_1. To the blank solution containing dilute sulfuric acid and the catalyst solution containing hydrogen peroxide, 1 - 2 drops of hydrogen peroxide solution were added. The mixture was then shaken to develop color. Subsequently, the absorbance reading was recorded as A_2. The content of Ti (ω) was calculated using the following equation:

$$\omega = \frac{A_2 - A_1}{m \times k} \times 5$$

k is the slope of the standard curve.

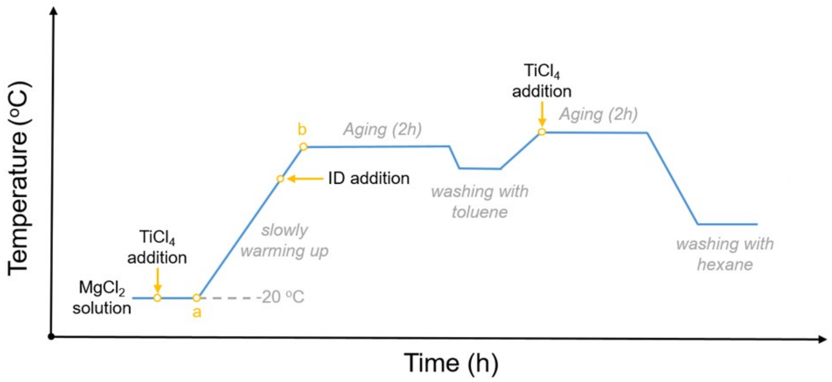

Scheme S1. Schematic description of the main steps upon ZNCs preparation.

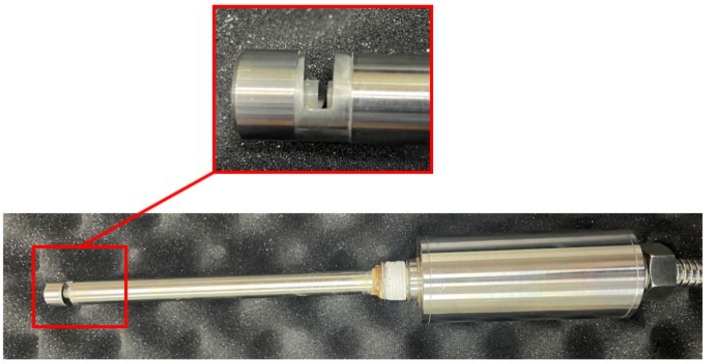

Figure S1. Details of in-line imaging probe.

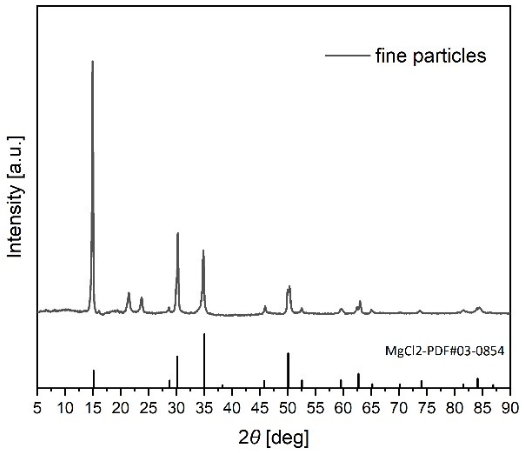

Figure S2. XRD result of black spots observed in Figure 2a, isolated by centrifugation. (In one experiment, we obtained 150 mg of white, insoluble solids from 160 mL of $MgCl_2$ solution by centrifugation. These solids accounted for around 2% of the initial $MgCl_2$ mass)

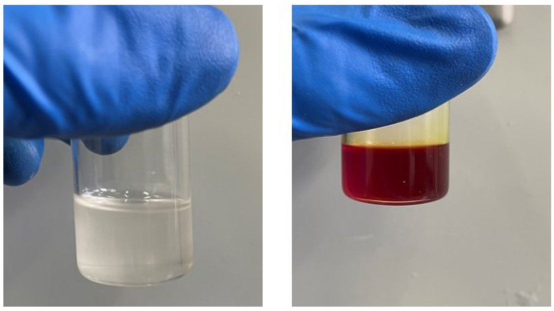

Figure S3. before adding $TiCl_4$ (left); after adding $TiCl_4$ (right).

Table S1. Pore structure parameters of Cat1 and Cat2.

catalyst	S_{BET} (m^2/g)	S_{ext} (m^2/g)	V_{total} (cm^2/g)
Cat1	259.9	300.8	0.19
Cat2	239.9	277.9	0.18

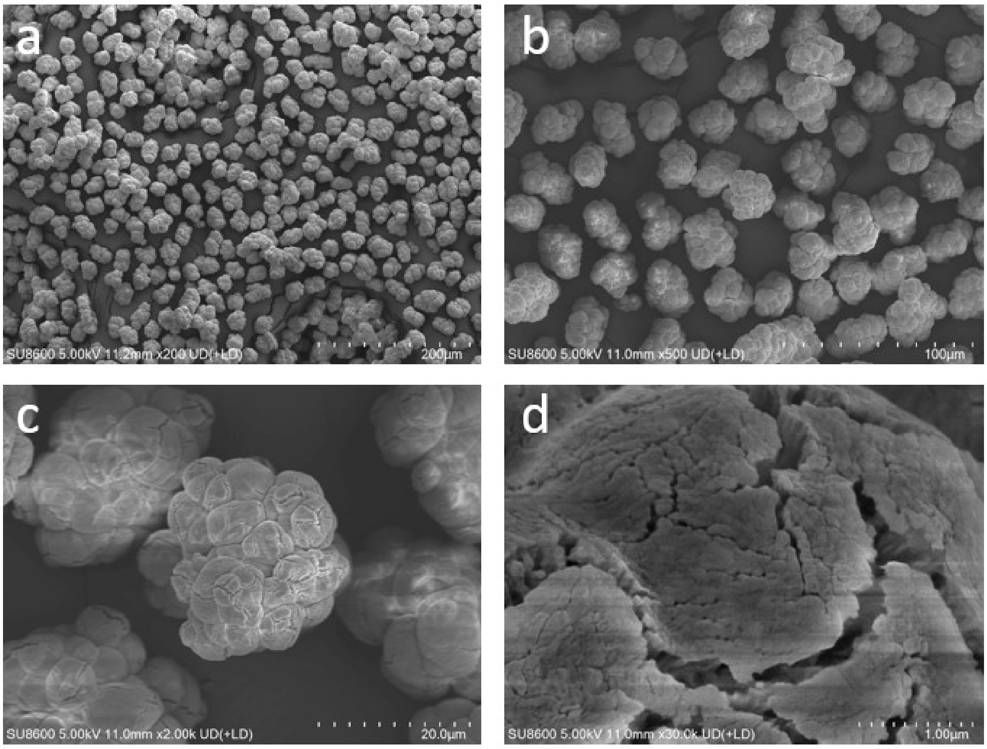

Figure S4. SEM micrographs of Cat2 particles collected at magnification 200x (a), 500x (b), 2000x (c) and 30000x (d).

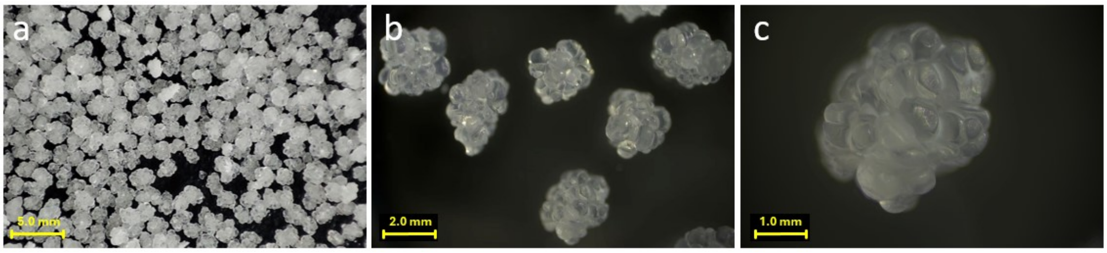

Figure S5. Photographs of resulting polypropylenes, taken from camera, b and c recorded from optical microscope.
